# Challenging the charge balance hypothesis: reconsidering buffer effect and reuptake of previously excreted organic acids by *Penicillium ochrochloron*

**DOI:** 10.1093/femsle/fnaa039

**Published:** 2020-03-04

**Authors:** D J Artmann, P Vrabl, R Gianordoli, W Burgstaller

**Affiliations:** Institute of Microbiology, University of Innsbruck, Technikerstrasse 25, 6020 Innsbruck, Österreich

**Keywords:** organic acid excretion, charge balance, reuptake, strain specificity, buffer capacity, *Penicillium ochrochloron*

## Abstract

*Penicillium ochrochloron* was used in the past for the leaching of zinc from a zinc oxide containing filter dust via excreted organic acids. Organic acid excretion by *P. ochrochloron* was stimulated by the addition of an extracellular buffer (2-(N-Morpholino)ethanesulfonic acid, MES; or zinc oxide, ZnO: ZnO + 2 H^+^ → Zn^2+^ + H_2_O). It was tested if the buffer stimulated excretion of organic acid anions is due to the necessity of an anion efflux across the plasma membrane to maintain electroneutrality by balancing the excretion of protons by the H^+^-ATPase. This charge balance hypothesis was previously postulated for *P. ochrochloron*. Two strains of *P. ochrochloron* were studied, which differed in growth parameters and amount of excreted organic acids. From the results, it was concluded that charge balance at the plasma membrane is not the main reason for organic acid excretion in these two strains of *P. ochrochloron*. Furthermore, the phenomenon of reuptake of excreted organic acids in the presence of about 100 mM of glucose is confirmed. It is suggested that the equilibrium between extracellular and intracellular organic acid anions may be maintained passively by a facilitated diffusion transporter.

## Teaser

This study disproves the charge balance hypothesis as the main reason for the excretion of organic acids in *Penicillium ochrochloron* and confirmes the reuptake of excreted organic acids despite the presence of glucose.

## INTRODUCTION

Organic acid production by filamentous fungi is of high relevance within the field of fungal biotechnology (Magnuson and Lasure 2004; Sauer, Porro and Mattanovich 2008). The most important of these organic acids are citric acid, gluconic acid, lactic acid, succinic acid and itaconic acid (Magnuson and Lasure 2004; Sauer, Porro and Mattanovich 2008). Despite their biotechnological relevance, many aspects such as trigger factors or even the basic question why fungi excrete these metabolites at all are still unclear.

Among the filamentous fungi *Penicillium ochrochloron* is the second-best studied fungus concerning organic acid excretion besides *Aspergillus niger*. We want, however, to emphasize that considering the high phenotypic plasticity and biochemical individuality of filamentous fungi (Foster 1949), the results reported here for *P. ochrochloron* cannot be transferred untested to other species or strains of filamentous fungi.

In *P. ochrochloron* the excretion of citrate and other organic acids was stimulated by and depended on extracellular parameters such as buffers (zinc oxide (ZnO), 2-(N-Morpholino)ethanesulfonic acid (MES), HEPES, phthalic acid; Burgstaller *et al*. 1992; Franz, Burgstaller and Müller 1993; Burgstaller, Zanella and Schinner 1994; Gallmetzer, Müller and Burgstaller 1998), a pH around 7 (Vrabl *et al*. 2012), nutrient exhaustion (Vrabl *et al*. 2012), the kind of limiting nutrient (Gallmetzer and Burgstaller 2002), osmolarity (Gallmetzer and Burgstaller 2001), potassium concentration (Gallmetzer and Burgstaller 2001), uncouplers (Gallmetzer and Burgstaller 2002), aeration (Gallmetzer, Meraner and Burgstaller 2002) and glucose concentration (Vrabl, Mutschlechner and Burgstaller 2008). Intracellular factors such as the Energy Charge (Vrabl, Mutschlechner and Burgstaller 2009), the activity of the plasma membrane H^+^-ATPase (Vrabl *et al*. 2017) and the activity of different complexes of the respiratory chain (Vrabl *et al*. 2017) were less influential.

No single hypothesis reported in the literature to explain organic acid excretion could explain all phenomena observed with *P. ochrochloron*, e.g. overflow metabolism (Foster 1949), charge balance hypothesis (Roos & Luckner 1984; Roos and Slavik 1987; Slayman, Kaminski and Stetson 1990), aggressive acidification hypothesis (Andersen, Lehmann and Nielsen 2009) or metal ion chelation (Odoni *et al*. 2017). For *P. ochrochloron* Burgstaller, Zanella and Schinner (1994) provided experimental and theoretical evidence that the charge balance hypothesis is relevant for organic acid excretion in this fungus further discussed in Vrabl *et al*. (2012). From the few references dealing with charge balance as a reason for organic acid excretion in filamentous fungi (Roos and Luckner 1984; Roos and Slavik 1987; Slayman, Kaminski and Stetson 1990; Burgstaller, Zanella and Schinner 1994; Vrabl *et al*. 2012) the methodologically most convincing one – because of applying electrophysiological measurements – is Slayman, Kaminski and Stetson (1990). In *P. ochrochloron*, however, electrophysiological measurements were not possible because of the smaller diameter of the hyphae. After all these hypotheses, it became the aim of the work presented here to further test our previous hypothesis about the role of charge balance at the plasma membrane as a cause for organic acid excretion in *P. ochrochloron* (Burgstaller, Zanella and Schinner 1994; Vrabl *et al*. 2012).

**Figure 1.**
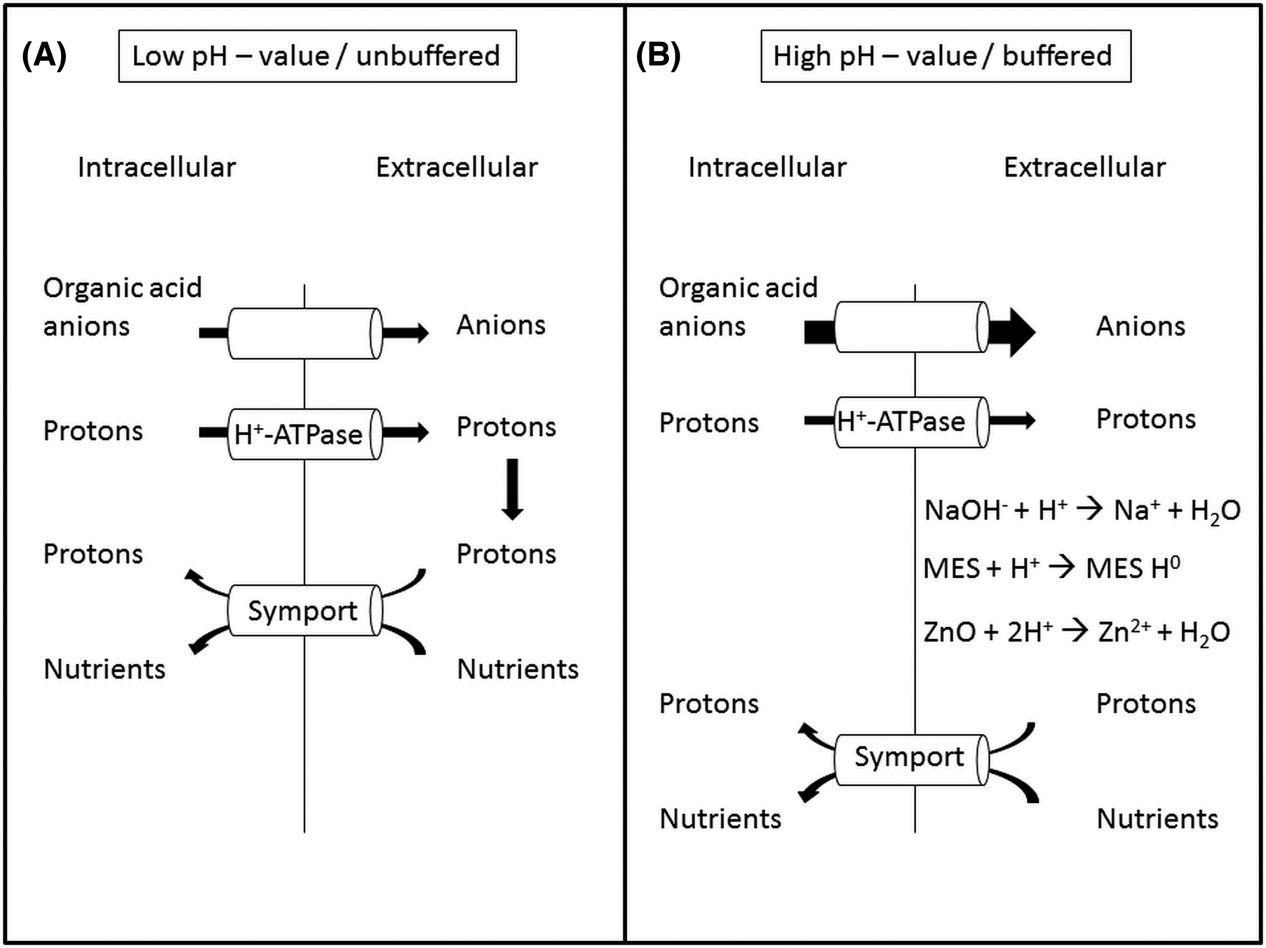
Cation and anion fluxes across the plasma membrane at **(A)**, low extracellular pH (unbuffered) with a sufficient charge balance by proton return and at **(B)**, high extracellular pH (buffered) with a hampered proton return because of the neutralizing effect of e.g. NaOH. In this case charge balance is provided by the excretion of organic acid anions.

Fluxes of cations and anions across the plasma membrane must be electrically equilibrated to avoid a too high membrane potential. The main cation flux across the plasma membrane is the excretion of protons by the plasma membrane H^+^-ATPase. At low extracellular pH, proton return by the uptake of nutrients via proton symports provides sufficient charge balance (Slayman, Kaminski and Stetson 1990). At neutral to alkaline extracellular pH, however, proton return becomes difficult, especially if excreted protons are neutralized, for instance, by NaOH. Charge balance for excreted protons must then be provided by other ion fluxes. This is all the more important because the proton flux mediated by the H^+^-ATPase is insensitive to extracellular pH and is thus not reduced at neutral to alkaline pH (Sanders 1988; Slayman, Kaminski and Stetson 1990). When proton return becomes difficult, charge balance for proton excretion is provided by the excretion of organic acid anions (Conway and Brady 1950; Gradmann *et al*. 1978; Sanders 1988; Slayman, Kaminski and Stetson 1990; Sigler and Höfer 1991; Burgstaller, Zanella and Schinner 1994; Rivetta, Kuroda and Slayman 2011; Vrabl *et al*. 2012). Charge balance by excretion of organic acids requires the organic acids being excreted as a negatively charged species. That this is a reasonable assumption was shown by thermodynamic calculations for citrate excretion (Burgstaller 2006). According to this charge balance hypothesis the excretion of organic acids must decrease at low pH (where proton return is possible and sufficient) and increase at neutral to alkaline pH (where proton return is hindered or impossible). The essence of the charge balance hypothesis is illustrated in Fig. 1.

In *P. ochrochloron* the stimulation of citrate excretion by zinc oxide was explained to be the result of the buffer effect of zinc oxide, because ZnO neutralizes protons to give zinc ions and water (ZnO + 2 H^+^ → Zn^2+^ + H_2_O; pH approximately 6; Burgstaller *et al*. 1992; Burgstaller, Zanella and Schinner 1994). Stimulation of organic acid excretion by ZnO thus falls within the charge balance hypothesis: The excretion of citrate anions provides charge balance for the excretion of positively charged protons across the plasma membrane when excreted protons are neutralized by ZnO and cannot flow back into the hyphae (Burgstaller, Zanella and Schinner 1994). A lower stimulation of citrate excretion was also achieved with 1 M MES/NaOH pH 6 (Burgstaller, Zanella and Schinner 1994), 1 M HEPES/NaOH pH 7.5 (Burgstaller, Zanella and Schinner 1994) or 1 M phthalic acid/triethanolamine pH 7.5 (Franz, Burgstaller and Müller 1993). The different stimulation of organic acid excretion by zinc oxide and by MES or HEPES is in line with the charge balance hypothesis: proton return is more strongly hindered with ZnO (protons are neutralized) than with MES and HEPES (protons are only bound to the buffer but are in principle still available for proton symports according to the mass action law). However, these high buffer concentrations resulted in a high extracellular osmolarity, which certainly gave rise to physiological side effects other than citrate excretion. A clear difference was demonstrated between *A. niger* and *P. ochrochloron* (Franz, Burgstaller and Müller 1993). Only with *P. ochrochloron* was the excretion of citrate stimulated by buffering the medium.

In the present work, we expanded the knowledge about the application of the charge balance hypothesis to *P. ochrochloron* and explored if this mechanism is the main reason for the excretion of organic acids in *P. ochrochloron* in the presence of an extracellular buffer. If the charge balance hypothesis is valid, then it should lead to a general increase of organic acid excretion independent of the strain used because charge balance is a purely physical reason. All the more ZnO should be a stronger stimulant than MES. We studied the effect of zinc oxide and MES on the excretion by *P. ochrochloron* with three approaches. First, we compared the stimulation by zinc oxide and a soluble buffer (0.2 M MES/NaOH pH 6), that kept the pH constant, but exhibited a lower extracellular osmolarity than in previous experiments with 1 M buffers (Burgstaller, Zanella and Schinner 1994). Second, we checked whether zinc oxide or MES also stimulated the excretion of organic acids other than citrate. And third, we compared two strains of *P. ochrochloron*, which differed in growth parameters and organic acid excretion (Vrabl *et al*. 2012). The rationale was that if the charge balance hypothesis is relevant then ZnO should in both strains be a stronger stimulant than MES.

In the course of these experiments, we confirmed the phenomenon of reuptake of some excreted organic acids in the presence of glucose – observed already in a different experimental context (Vrabl *et al*. 2012). We provide a hypothetical mechanism for this organic acid reuptake in the presence of glucose. As we discuss in this work, organic acid reuptake in the presence of glucose still lacks a plausible explanation. It may be that organic acid reuptake in the presence of glucose does not serve as an additional carbon source but is due to an entirely different reason.

## MATERIAL AND METHODS

### Organism

As described in Vrabl, Mutschlechner and Burgstaller (2008) a fungal strain was isolated from soil of the copper works Montanwerke Brixlegg (Austria) in 1989 and identified as *Penicillium simplicissimum* by the Centraalbureau voor Schimmelcultures (CBS, the Netherlands). After cultivation since the last 20 years a re-identification from a frozen culture of that time and the actual laboratory strain was arranged. This re-identification showed that both strains are *P. ochrochloron*, a fungus closely related to *P. simplicissimum* (Tuthill, Frisvad and Christensen 2001). Furthermore, there was amongst others, a difference in the range of secondary metabolites, so that the original wild-type strain was called CBS 123823 and the current laboratory strain was called CBS 123824. All previous publications had to be corrected since that time. Both strains of the filamentous fungus *P. ochrochloron*, CBS 123823 and CBS 123824, were used in this work.

### Media composition

The medium for both strains in the ZnO (zinc oxide) approach consisted of 395.54 mmol L^−1^ glucose monohydrate, 6.19 mmol L^−1^ (NH_4_)_2_SO_4_, 12.37 mmol L^−1^ NH_4_Cl, 1.59 mmol L^−1^ MgSO_4_.7H_2_O, 5.74 mmol L^−1^ KH_2_PO_4_ and 9.90 mL L^−1^ double concentrated trace element solution consisted of 3.60 mmol L^−1^ Fe(II)SO_4_.7H_2_O, 2.72 mmol L^−1^ Mn(II)SO_4_.1H_2_O, 2.94 mmol L^−1^ ZnCl_2_, 0.40 mmol L^−1^ Cu(II)SO_4_.5H_2_O, 4.08 mmol L^−1^ CaCl_2_.2H_2_O and 0.39 mmol L^−1^ CuSO_4_ (medium from the end report of the Austrian Science Fund project P15491); sucrose was replaced by glucose; Mutschlechner 2006). Per 100 mL Erlenmeyer flask 340 mg ZnO/20.4 mL were added. The 20.4 mL resulted from 20 mL medium and 400 µL inoculum. For the MES (2-(N-morpholino)ethanesulfonic acid) approach the same medium for both strains was used but instead of zinc oxide the medium was buffered with 200 mmol L^−1^ MES. Sugar, salts and the MES module were autoclaved separately. In the unbuffered approach neither ZnO nor MES was used. The missing volume caused by the MES module was compensated by deionized water in the ZnO and unbuffered approach. The trace element solution was sterilized separately by filtration. The pH values were adjusted to 6.5 with 5 M NaOH so that a uniform pH value in the range of 6.1–6.4 was achieved after inoculation.

### Spore suspension

The substrate consisted of 50 g rye grains mixed in a 500 mL Erlenmeyer flask with 50 mL deionized water. After sterilization at 121°C, the content of the flask was inoculated with 1 mL thawed spore suspension (3.2 × 10^8^ spores/mL) of *P. ochrochloron* under sterile conditions and carefully mixed. After incubation at 30°C for 7 days, the spores were washed away with 50 mL of a sterile 0.05% Tween80 solution and aliquoted at 1 mL each in Cryotubes. The spore density was counted in a thoma chamber and afterwards frozen at −20°C until further use.

### Organism cultivation

Each Erlenmeyer flask was filled with 20 mL medium and subsequently inoculated with 0.4 mL spore suspension to a final concentration of 10^6^ spores per mL. The flasks were cultivated in a climatic chamber at 30°C at a relative humidity of approximately 55% with 350 rpm on a rotary shaker. Since the evaporation loss of the Erlenmeyer flasks was 0.3% on average after 36 h of incubation, 0.3% after 48 h, 0.5% after 72 h and a maximum of 0.8% after 96 h due to automatic humidification, no evaporation correction was made for the duration of the experiment.

### Sampling and analytics

The time points for sampling were 0, 36, 48, 72, 96, 120, 144, 168 and 192 h. Each time the whole culture broth was taken from twelve parallel cultures with the exception of the sampling point 0 h, where the culture broth was taken from six parallels. A sample (150 µL) was taken immediately from each Erlenmeyer flask for microscopy (Leitz Diaplan). The culture broth was then vacuum-filtrated (MN 640 m, Nr 43, Macherey-Nagel) in a multi-sample-vacuum filtration system (Millipore, MERCK) and the filter cake washed with 5 mL cold deionized water. The pH value was measured before filtrate preparation (0.45 µm, 25 mm, OPTI-FLOW regenerated cellulose syringe filter from WICOM) for high-performance liquid chromatography (HPLC). All filter cakes were dried for 12 h at 105°C and samples with zinc oxide were then incinerated to estimate the anorganic amount for 3 h at 540°C. Organic acids (oxalate, citrate, pyruvate, malate, succinate and fumarate) were measured by HPLC as described in Womersley, Drinkwater and Crowe (1985), (Waters; column Aminex HPX 87H from BIORAD, 515 HPLC pump, 717plus Autosampler, 2487 Dual λ Absorbance Detector, flow rate 0.6 mL*min^−1^, temperature 41°C, 4 mN H_2_SO_4_; injection volume 20 µL, λ = 210 nm). Glucose was analyzed with the glucose assay from Megazyme International Ireland Ltd. and measured photometrically (λ = 510 nm, Ultrospec 2000, Pharmacia Biotech). Ammonium was analyzed according to the macroscale method after Rhine *et al*. 1998. Phosphate was analyzed with a modified method after Illmer 1996.

## RESULTS AND DISCUSSION

The aim of this work was to test whether or not the charge balance hypothesis explains organic acid excretion in *P. ochrochloron* as stated by Burgstaller, Zanella and Schinner (1994). To achieve this, we chose an experimental setup as similar as possible to the one previously published (Burgstaller, Zanella and Schinner 1994), in which ZnO was used as triggering agent. Even one strain, *P. ochrochloron* CBS 123824, was identical to the strain used in that former study, as it was a frozen aliquot of the spore suspension from that time. The second strain in the current work was the wild-type strain *P. ochrochloron* CBS 123823. If the charge balance hypothesis holds true zinc oxide should stimulate organic acid excretion in both of these strains and zinc oxide should have been a stronger stimulant than MES.

The obtained results do not support that charge balance at the plasma membrane is the main reason for organic acid excretion in the tested strains *P. ochrochloron* CBS 123823 and CBS 123824 because (i) with CBS 123823 MES was for all acids a stronger stimulant for organic acid excretion than ZnO and (ii) with CBS 123824 zinc oxide was a better stimulant only at the beginning of the exponential growth phase.

### Media, growth characteristics and morphology

**Figure 2.**
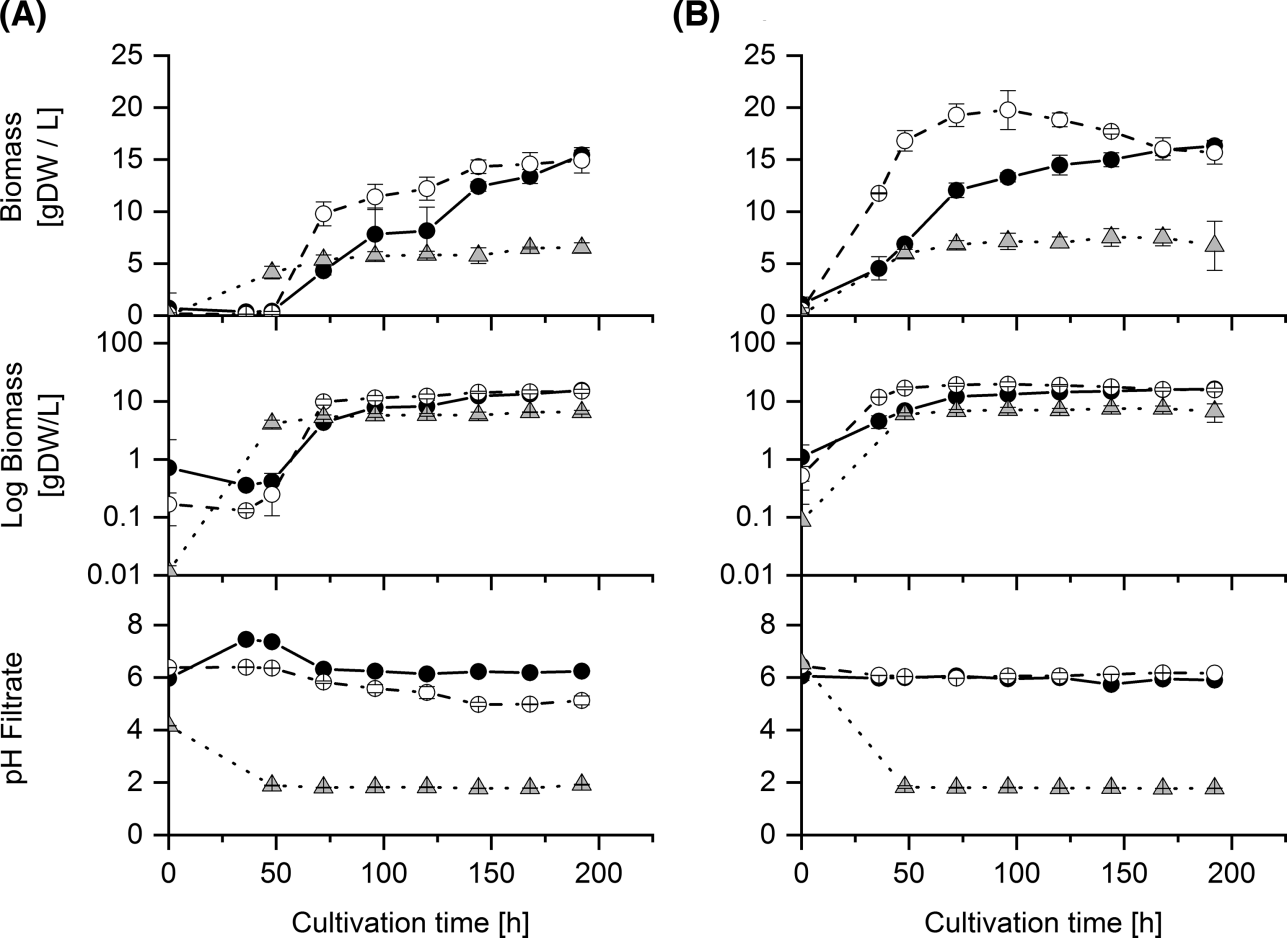
Growth characteristics of shake flask cultures of two *Penicillium ochrochloron* strains with three different experimental settings (MES, ZnO, unbuffered). **(A)**, CBS 123823, **(B)**, CBS 123824; (○) MES (●) ZnO (△) unbuffered; Biomass concentration, logarithmic biomass and pH values of the filtrates. Data are means of typically 12 independent cultivations with exception of the sampling point 0 h, where the data represent the mean of 6 independent cultivations.

Three different media – and thus potential stimulation conditions – were used in this work, which were (i) the basic medium buffered with 0.2 M MES with a buffering capacity of 0.1 M H^+^ at pH 6 (pH ≈ pKa), (ii) a zinc oxide buffered basic medium (0.205 M) with a buffering capacity of 0.4 M H^+^ (ZnO + 2 H^+^ → Zn^2+^ + H_2_O) at pH 6 and (iii) an unbuffered basic medium. This unbuffered medium was selected as a control to (i) observe how the excretion behaves when no extracellular buffer affects the culture and (ii) because this was in our opinion the only feasible control as any pH-controlled scenario will inevitably neutralize protons. From Burgstaller, Zanella and Schinner (1994), we knew that in an unbuffered medium the pH decreased rapidly to about 2, very little biomass was formed and almost no organic acids were excreted. Because of this we were not interested in the initial growth phase but rather in later growth phases and did not take samples during this period. Although growth stopped when the pH fell to about 2, during the short growth period specific excretion of organic acids could be calculated [µmol (g DW)^−1^]. A pH of 6 could be maintained in both buffered media throughout the whole cultivation period (Fig. 2; with the exception of CBS 123823, which showed an initial spike due to the solubilization of ZnO and the slower growth of this strain, as well as an pH decrease to about five due to insufficient buffering capacity of 0.2 M MES; Fig. 2).

**Figure 3.**
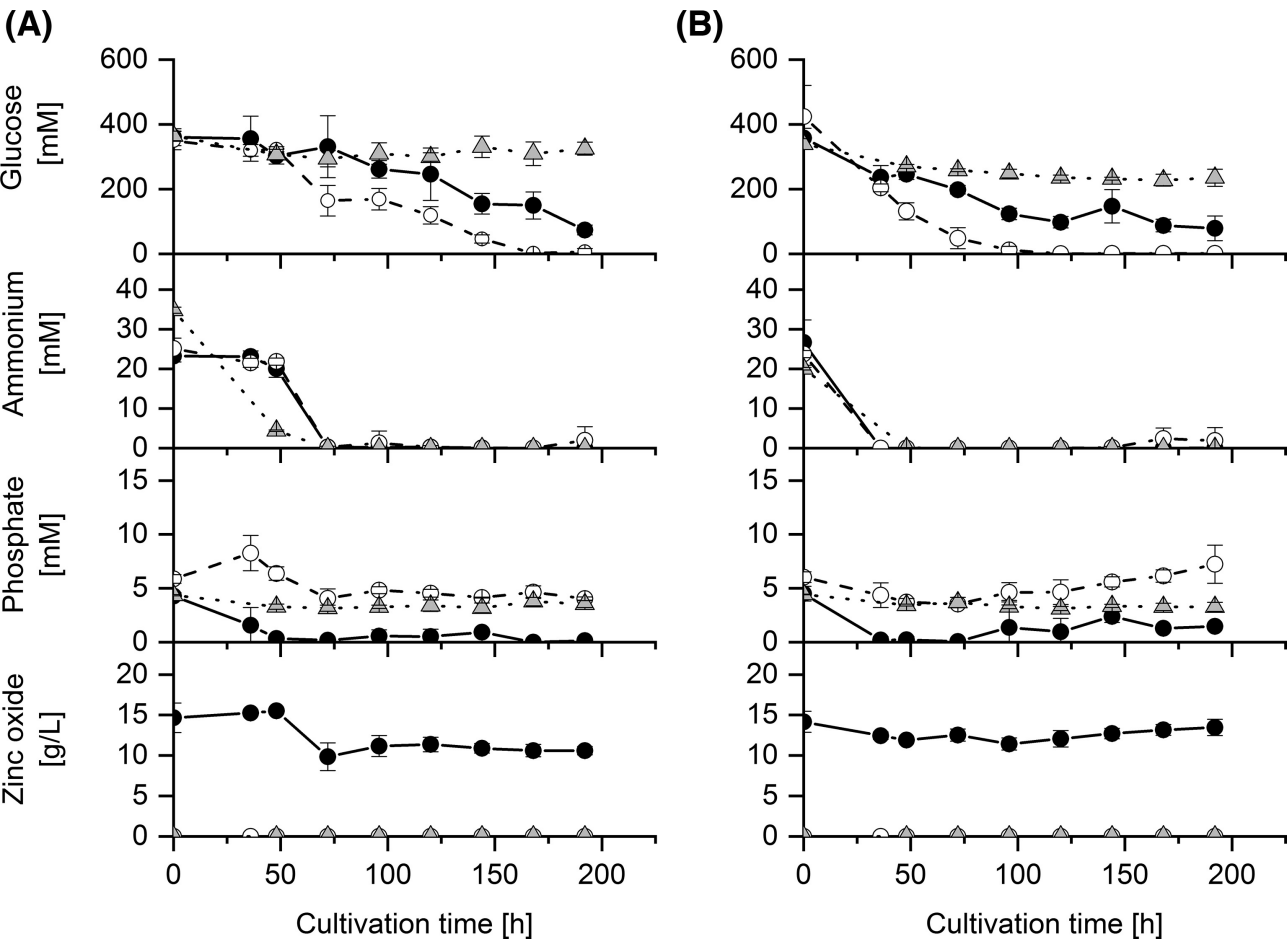
Residual nutrient concentrations and the residual zinc oxide concentration of two *Penicillium ochrochloron* strains of three different experimental settings (MES, ZnO, unbuffered). **(A)**, CBS 123823, **(B)** CBS 123824; (○) MES (●) ZnO (△) unbuffered; glucose, ammonium and phosphate and zinc oxide; data are means of typically 12 independent cultivations with exception of the sampling point 0 h, where the data represent the mean of 6 independent cultivations.

The strain CBS 123823 exhibited a longer lag phase than strain CBS 123824 (Fig. 2). Both strains exhibited higher specific growth rates with MES compared to ZnO (CBS 123823: µ = 0.15 h^−1^ with MES and µ = 0.10 h^−1^ with ZnO; CBS 123824: µ = 0.07 h^−1^ with MES and µ = 0.04 h^−1^ with ZnO (Fig. 2). Strain CBS 123824 achieved – at least with MES – a higher biomass concentration (maximum of 19.3 g DW/L) than strain CBS 123823 (maximum of 14.9 g DW/L) (Fig. 2). With strain CBS 123824 and MES, biomass decreased from a maximum of 19.8 g DW/L when glucose was exhausted to 15.7 g DW/L at the end of cultivation time. In the unbuffered controls, the pH decreased to 1.9 and only little biomass (maximum of 6.5 g DW/L) was formed (Figs. 2 and 3). The exponential growth phase ended when ammonium was consumed (at 72 h with CBS 123823; at the latest at 48 h with CBS 123824).

Concerning glucose consumption, it is striking that with ZnO as a buffer in cultures of both strains, at least 74 mmol/L of glucose remained in the medium at the end of cultivation (Fig. 3). With strain CBS 123824 and MES as buffer, glucose was exhausted earlier (120 h; Fig. 3) than with strain CBS 123823 and MES (168 h; Fig. 3). The same was true for ammonium exhaustion (72 h for strain CBS 123823; 36 h for strain CBS 123824; Fig. 3). In MES buffered cultures phosphate was still present at the end of cultivation with both strains, whereas in ZnO buffered cultures phosphate had disappeared at about 50 h of cultivation with both strains (Fig. 3). If we consider phosphate exhaustion during increased organic acid excretion as a change in the environment, this triggering nutrient limitation would fit to the overflow metabolism hypothesis rather than to the charge balance hypothesis. On the other hand, phosphate exhaustion could also be possibly due to the abiotic effect of the precipitation of phosphate as insoluble zinc phosphate in ZnO cultures. Anyway, more ZnO was solubilized with strain CBS 123823 than with strain CBS 123824 (Fig. 3). Phosphate detected at later points in time may be due to the release of phosphate by autolysis.

Altogether these results confirmed that both strains differed in their growth characteristics, where CBS 123824 showed a faster and higher biomass formation compared to CBS 123823.

Although strain CBS 123824 had consumed more glucose and had formed more biomass than strain CBS 123823 at the end of the exponential phase, the biomass yield (g DW/g glucose) was nearly identical at this time point (0.28 and 0.32).

Concerning the morphology of both strains, there were clear differences within the three different conditions (MES, ZnO and unbuffered) (Fig. 5): With MES, the pellets were clearly the largest (diameter ∼ 50 µm) and the hyphae were longer. With zinc oxide, the pellets were several times smaller in diameter (∼ 0.2 µm) and had extremely short hyphae, but there were more pellets than in the MES and the unbuffered approach. In unbuffered cultures the pellet size (∼ 25 µm) was between those from ZnO and MES-buffered cultures.

### Effect of MES and ZnO on organic acid excretion

**Figure 4.**
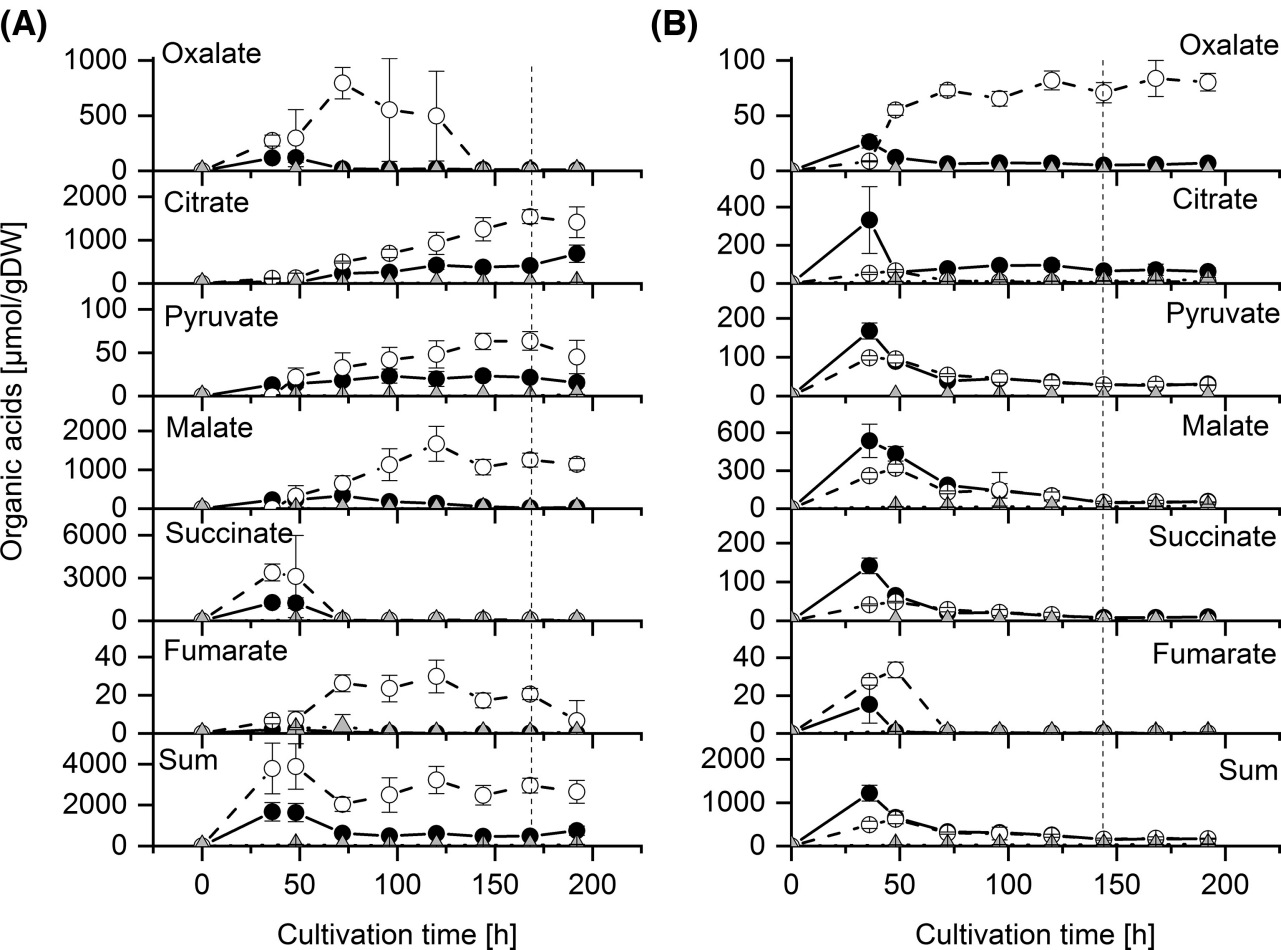
Excreted organic acids in shake flask culture cultivations of two *Penicillium ochrochloron* strains of three different experimental settings (MES, ZnO, unbuffered). a) CBS 123 823, b) CBS 123 824; (○) MES (●) ZnO (△) unbuffered; the dashed vertical line shows the time point of C-depletion of the MES approach. Data are means of typically 12 independent cultivations with exception of the sampling point 0 h, where the data represent the mean of 6 independent cultivations.

The wild type strain CBS 123823 excreted much more organic acids than strain CBS 123824 – both with ZnO (1.7 times more) and MES (7.2 times more) (Fig. 4). The stimulation of especially citrate excretion by an extracellular buffer is a distinct difference to citrate excretion by *A. niger*, whose maximum citrate excretion is at a pH of 2–3 (Franz, Burgstaller and Müller 1993; Ruijter, van de Vondervoort and Visser 1999). In unbuffered cultures, where the pH dropped from pH 4.2 to pH 1.9, excretion of organic acids per gram of dry weight was more than 20 times lower with the strain CBS 123823 and between 20 (ZnO) and 150 (MES) times lower with the strain CBS 123824 (Figs 2 and 4).

**Figure 5.**
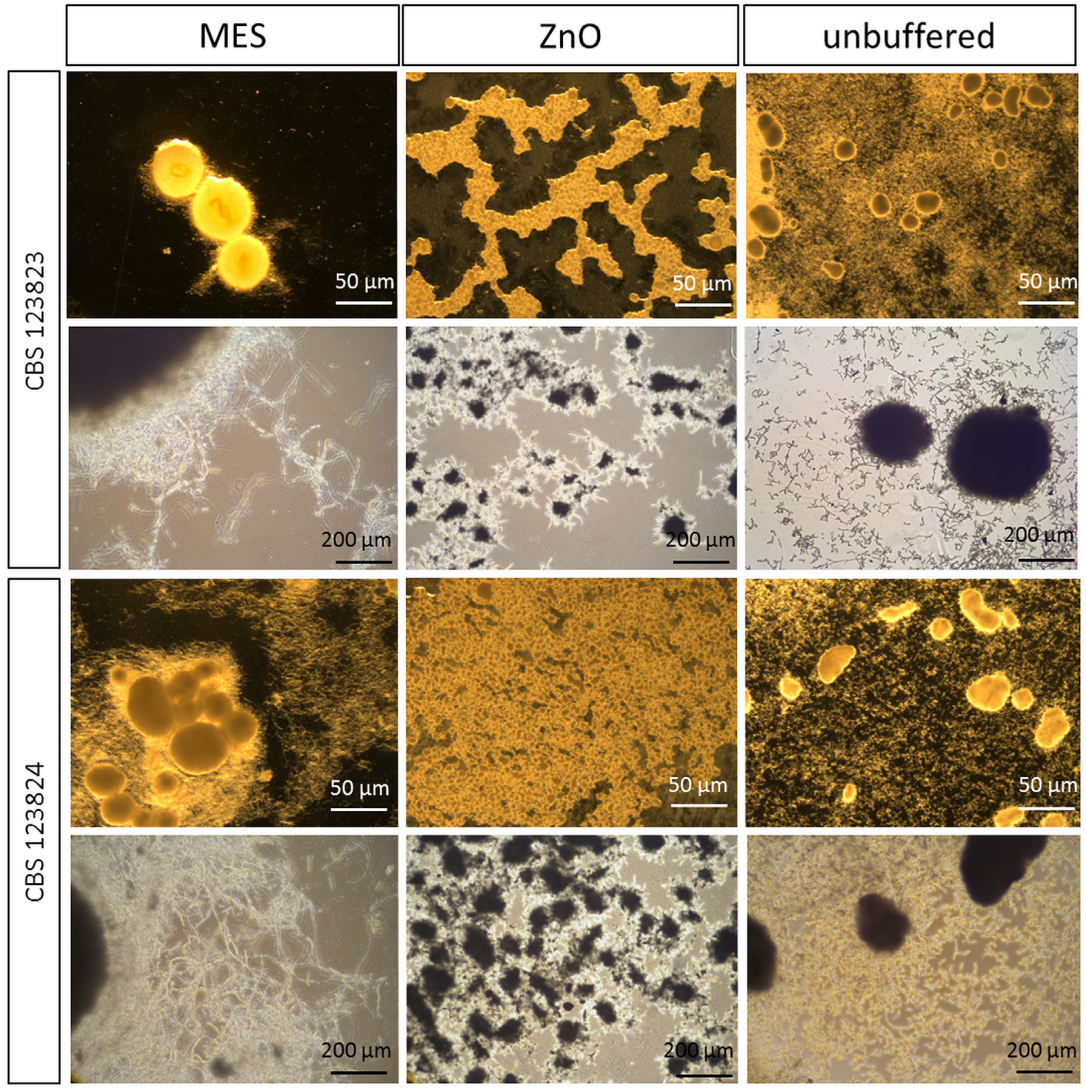
Microscope images of two *Penicillium ochrochloron* strains (CBS 123823 and CBS 123824) after 96 hours cultivation time of the MES approach, the ZnO approach and the unbuffered approach.

A stronger stimulation of organic acid excretion by ZnO than by MES was only observed during the exponential growth of strain CBS 123824 (except oxalate and fumarate; Fig. 4). In all other cases MES was a better stimulant than ZnO (Fig. 4). This is particularly clear with strain CBS 123823 and MES (Fig. 4). This was unexpected and cannot be attributed to the decreasing pH in CBS 123823 cultures, since the organic acid excretion in *P. ochrochloron* is higher at pH 7 than at pH 5 (Vrabl *et al*. 2012). That MES was a better stimulant for organic acid excretion than ZnO contradicts the charge balance hypothesis as postulated for *P. ochrochloron* (see Fig. 1; Burgstaller, Zanella and Schinner 1994).

From the examination of single organic acids, the following picture emerged (Fig. 4): Citrate, pyruvate and malate increased during post exponential growth, whereas oxalate, succinate and fumarate decreased (Fig. 4). With strain CBS 123824, the picture was more heterogeneous (Fig. 4). Fumarate and oxalate were found in higher amounts with MES than with ZnO. With citrate, malate and pyruvate it was the other way round. ZnO stimulated citrate excretion fivefold, malate and pyruvate excretion was almost stimulated twice by ZnO (Fig. 4).

With both buffers, both strains excreted not only citrate, but also pyruvate, malate, fumarate, succinate and oxalate (Figs. 2 and 4). Thus both buffers led to the excretion of a similar pattern of organic acids, which was also found with NaOH in batch and continuous bioreactor culture (Gallmetzer and Burgstaller 2001; Vrabl *et al*. 2012). This means that the kind of buffer does not influence the pattern of excreted organic acids. However, the kind of buffer influences the amount of excreted organic acids – and this in addition depended on the strain (compare the scales of the *y*-axes in Fig. 3). For this different metabolic behavior between the two strains, we have no reasonable and straightforward causal or functional explanation.

### Reuptake of organic acids in the presence of glucose

In these experiments and also in earlier work (Vrabl *et al*. 2012), we noticed the reuptake of excreted organic acids, although there was still sufficient glucose in the medium. Reuptake of excreted organic acids was not observed before 48 h of cultivation (not considering the early growth phase of the control where almost no organic acids were excreted). With strain CBS 123823 and MES, oxalate, malate, succinate and fumarate were taken up again before glucose depletion (at 168 h of cultivation). With strain CBS 123824 and MES, with exception of oxalate, all organic acids were taken up before glucose was exhausted (at 120 h of cultivation). With ZnO as a buffer – and glucose still present – a slight uptake of oxalate, malate and succinate was measured in CBS 123823, whereas a reuptake of all excreted organic acids was determined in CBS 123824. On a closer look at the two strains, a reuptake was observed as follows: oxalate with strain CBS 123823, citrate with strain CBS 123824, malate with both strains, pyruvate with CBS 123824, succinate with both strains, and fumarate with both strains. As with the excretion of organic acids, also the reuptake in the presence of glucose turned out to be strain specific.

The reuptake of organic acids after exhaustion of glucose can be explained as the usage of a still available carbon source. There is, however, no straightforward explanation for the reuptake of excreted organic acids in the presence of glucose. Vrabl *et al*. (2012) observed a reuptake of organic acids by CBS 123824 in the presence of glucose at pH 5 in bioreactor culture after the exhaustion of ammonium. Simultaneously, a short increase in the pH value was observed. This could indicate organic acid uptake via a proton symport mechanism.

An uptake of organic acids was reported by Sousa, Mota and Leão (1992) and Saayman *et al*. (2000), both of which were also discussed in Vrabl *et al*. (2012). In Sousa, Mota and Leão (1992), however, reuptake was observed in yeasts and the acids that were taken up, were not excreted but added at the start of the cultivation. This was therefore an uptake, but not a reuptake of previously excreted acids. It is in our opinion a physiologically different situation whether organic acids are added at the start of the culture (because the fungus adapts to the uptake of theses acids from the beginning of the culture) or these organic acids are first excreted by the fungus and only later taken up.

Accordingly, veritable reuptake, apart from our observation, are scarcely found in the literature. However, a recent ^13^C-tracer study with *P. chrysogenum* (Schmitz *et al*. 2013) revealed a permanent influx and efflux of the targeted organic acids despite the presence of high external sugar concentrations. Further, Roos and Luckner (1984) by using the isotope dilution assay found that there was a simultaneous net efflux and influx of citrate in *Penicillium cyclopium*, even when glucose was still present. In early growth phases efflux dominated, whereas in later growth phases uptake was predominant.

In *P. ochrochloron* there is substantial support for the hypotheses that citrate efflux is a passive transport process (see thermodynamic calculations in Burgstaller 2006), mediated by a transport protein (Gallmetzer, Müller and Burgstaller 1998) being not a reversed citrate uptake system (Gallmetzer, Müller and Burgstaller 1998). Therefore, we suggest the hypothesis that efflux and uptake of citrate (and other organic acid anions) is mediated in *P. ochrochloron* by a facilitated diffusion transporter. That facilitated diffusion transporter can be reversed is common knowledge (Kell 2019). Passive transport means that the intra- and extracellular concentrations of the transported ion species must be in equilibrium with the driving force. When the driving force changes, intra- and extracellular citrate concentrations must also change accordingly and a reuptake may occur. A transition from a higher to a lower membrane potential could be such a change in driving force and thus trigger the reuptake of citrate.

The reuptake of previously excreted carbon containing metabolites despite still available easily utilizable carbon sources seems to be more frequent in fungi than previously thought (e.g. Andersen, Lehmann and Nielsen 2009; Olejníková *et al*. 2011; Vrabl *et al*. 2012; Schmitz *et al*. 2013). This reuptake in the presence of glucose possibly does not serve as the uptake of an additional carbon source but might serve another, yet unknown metabolic functions.

## CONCLUSIONS

This study (i) tests the hypothesis of charge balance at the plasma membrane as the main reason for organic acid excretion in *P. ochrochloron* and (ii) confirms the reuptake of excreted organic acids in the presence of glucose. For this purpose, two *P. ochrochloron* strains, CBS 123823 and CBS 123824, were compared regarding their organic acid excretion in the presence of the extracellular buffers MES and ZnO. The physiology of the two strains was distinctly different concerning such fundamental metabolic properties as growth and organic acid excretion. In both strains and with most of the organic acids MES was a better stimulant for organic acid excretion than ZnO. In consequence, albeit charge balance at the plasma membrane may be one reason for organic acid excretion in *P. ochrochloron*, it cannot be the main reason.

This conclusion, however, is only valid for these two strains of *P. ochrochloron* and cannot be extended to organic acid excretion by other filamentous fungi. This extreme strain specificity is in line with a multitude of observations from filamentous fungi that any metabolic activity is subjected to a strong variability and phenotypic plasticity, which calls for an extensive physiological characterization at the strain level in order to understand and exploit biotechnologically a specific physiological property (Foster 1949; Rayner, Watkins and Beeching 1999; Vrabl *et al*. 2017).

The reuptake of excreted organic acids in the presence of glucose indicate that organic acid excretion and reuptake could fulfill a still unknown metabolic function in *P. ochrochloron*, e.g. balancing intracellular metabolite pool concentrations between different metabolic pathways or balancing extra- and intracellular concentrations or organic acids as a response to changing environmental conditions.
